# Fabrication of silver nanoparticles/gelatin hydrogel system for bone regeneration and fracture treatment

**DOI:** 10.1080/10717544.2020.1869865

**Published:** 2021-02-01

**Authors:** Xingwen Han, Jingjing He, Zhan Wang, Zhongtian Bai, Peng Qu, Zhengdong Song, Wenji Wang

**Affiliations:** a Department of Orthopaedics, The First Hospital of Lanzhou University, Lanzhou, China; b Department of Liver Disease, Lanzhou University Second Hospital, Lanzhou, China; c Department of Orthopaedics, Gansu Provincial Hospital, Lanzhou, China; d Key Laboratory of Biotherapy and Regenerative Medicine of Gansu Province Lanzhou, The First Hospital of Lanzhou University, Lanzhou, China

**Keywords:** AgNPs, gelatin, bone fracture

## Abstract

The present work aims to examine the effect of gelatin on the stabilization of silver nanoparticles (AgNPs) and their use in healing the bone fracture. AgNPs-loaded Gel hydrogels (AgNPs/Gel) were fabricated under sunlight using gelatin (Gel) as stabilizing agent. The characterization of the synthesized hydrogels was performed with the help of techniques such as UV-visible spectroscopy (UV-Vis) and high-resolution transmission electron microscopy (HR-TEM). Furthermore, the results of cell cytotoxicity confirmed that the AgNPs/Gel hydrogels are nonhazardous to osteoblasts. The outcome of cell fixation with AgNPs/Gel hydrogels after an incubation period of five days exposed the improved survival and spreading of osteoblasts cells on the prepared AgNPs/Gel hydrogels. Moreover, the AgNPs/Gel hydrogel nanostructures displayed their ability in modulating bone fracture healing, which suggests their potential use in nursing care.

## Introduction

Hydrogels are cross-linked to the bond of hydrophilic polymers that swell in the water-mediated medium. It exhibits effective division of biocompatible materials and it can be easily adopted for the production of mechanical, biological and chemical properties which resemble the native extra-cellular matrix and exhibit greater permeability toward nutrient products, oxygen molecules and other materials that are soluble in water. Modern advancements for developing a novel intelligent nanocomposite matrix of hydrogel for various applications such as traumatic wounds, dental fields, diabetic ulcers, antibacterial activity and in orthopedic fields are being discovered (Tian et al., 2007; Atiyeh et al., 2007; Mishra et al., 2008). The composition of implants is more concentrated to yield materials that are highly efficient containing biocompatibility, implant interface, osteo-induction, bio-active and effective passive integration at bone (Lu et al., 2007; Ma & Zhang, 2008; Zhang et al., 2016]. Recently, a lot of investigations are being developed involving AgNPs and alloy-dependent hydrogel matrix for the treatment of bone fracture, biomedical applications and dental field (Biffis et al., 2003; Si et al., 2013). In the recent years, fracture healing and bone repair are the postnatal routes that represent various ontological procedures occurring during the embryonic development of the framework. Although, bone fracture unify through quick surgical or conventional therapy, a significant proportion of fractures (5–10%) lack in healing and may end up being nonunion. In addition, for the nonunion cases, conventional methods are usually persistent and need a prolonged time period. Hence, exploration of latest strategies for the development of fracture repair are being continued (Zhang et al., 2016).

Nowadays, biopolymers receive greater attention in different areas like catalytic reactions, electrochemistry and specifically in biomedical applications (Selvam et al., 2017; Balaji et al., 2018). Gelatin is majorly applied in drug delivery systems owing to their strong biocompatibility. In detail, in order to enlarge the cell viability, a multiple layered composite coating was applied on Ag substrates (Cai et al., 2005). The results of an earlier report described the synthesis of gel-loaded nanotube substances to protect the biological activity of BMP2 (Hu et al., 2012). The individually synthesized multi-layer structure functioned as a biomimetic extracellular template that sustained the biomolecule release rate. However, no one has developed till now the unique fabrication of nanostructures tailored morphologically with bio-composited co-polymeric gel hydrogel to manage hard tissue nursing care and to accelerate bone fracture healing as per our knowledge.

On the other hand, NPs are demonstrated to exhibit important biological feature like antimicrobial activity to use in daily life (Khashan et al., 2014; Ismail et al., 2018). AgNPs showed important properties such as SPR, high extinction coefficient, and anti-bacterial that are comparatively less toxic than bulk form. AgNPs have exhibited various applications in day-to-day life. For example, AgNPs infused storage containers, medical devices coated with AgNPs were already developed to reduce infections in hospitals because of their anti-bacterial property (Al-Shmgani et al., 2017; Mehrabani et al., 2018; Pinto et al., 2018; Nakamura et al., 2019). AgNPs are an important additive in majority of health care products such as in footwear, bandages, antibacterial melamine foams and other house hold items because of their unique ability to fight against infectious diseases, by reducing the bacterial growth, germs and mold (Durán et al., 2016). Since, the AgNP-based compounds are being used as antibacterial agents in several biomedical applications, new possible investigations have begun to check the use of Ag NPs in orthopedic treatment applications (Brennan et al., 2015).

The production of NPs involving chemical and physical approaches are cost-effective and the used chemicals for synthesis may exhibit high reactive nature with potent environmental threats (Zare et al., 2017). Therefore, the involvement of such chemicals in the process of NP production limits their clinical and therapeutic uses. Owing to these limitations, the development of processes that are safe and sustainable to environment are highly recommended (Khatami et al., 2015). Therefore, approaches based on green chemistry are being considered in the NP production since past few years. The green chemistry method involves low-cost production of NPs without any involvement of toxic substances that may cause harm to the environment as well as human health (Tamuly et al., 2013).

The present study reports a new approach to produce AgNPs loaded hydrogels, which were fabricated effectively by a simple method under sunlight using gelatin as stabilizing agent. Furthermore, the cytotoxicity of AgNPs/Gel hydrogels is studied toward osteoblasts to know their application in fracture treatment, which showed the nontoxic nature of prepared hydrogels. The outcome of cell fixation with AgNPs/Gel hydrogels after an incubation period of five days exposed the improved survival and spreading of osteoblasts cells on the prepared AgNPs/Gel hydrogels.

## Materials and methods

### Fabrication of gelatin-loaded AgNPs hydrogel

The fabrication of AgNPs loaded with gelatin hydrogel was performed by following an earlier reported procedure (Thanh et al., 2018). Briefly, 3% wt concentrated aqueous solution of gelatin combined with 1% wt concentrated silver nitrate solution was exposed to diffused sunlight for various durations like 1 h, 3 h, 6 h and 12 h.

### Immunocytochemical staining

Bone-specific protein molecules like RUN X2, osteocalcin and osteopontin were analyzed using immunofluorescence microscope. The cells were fixed with 5% PFA and permeabilized with 0.5% Triton X-100 in phosphate buffer solution (PBS). Antibodies of osteopontin (mouse antibodies), osteocalcin along with RUN X2 (goat antibodies) were diluted at a ratio of 1:100 and added to the cells following with an incubation at a temperature of 5 °C overnight.

### Cell viability assay (live/dead)

Using the live/dead viability kit, the viability of sample cell was determined. According to the standard protocol, the cell-laden hydrogels were cleaned using PBS and seeded into osteoblast cells for performing the live/dead assay. The administrated cells were subjected to incubation in 4 µM concentration of Ethidium homodimer solution and 2 µM concentration of calcien amand for 30 min in order to examine their morphology. The fluorescent microscopy was used to visualize the prepared samples.

### DAPI/actin staining and attachment of cell

The wells seeded with cells were cleaned with PBS followed by incubation for 5 min in dark and then the cells were examined under fluorescent microscope. In addition, DAPI was used as nuclear stain.

### Analysis of alkaline phosphatase

SensoLyte pNPP alkaline (ALP) kit was utilized to examine the ALP activity as an exchange of p- nitrophenyl to p-nitrophenol because of cellular extract activity. After washing the samples with lysis buffer and Dulbecco’s PBS, the samples were mixed well with hydrogel. All the samples of hydrogel were incubated in an ice-cold water bath followed by centrifugation for 10 min at 5 °C temperature for precipitating material and cellular waste.

### Assay of calcium

To determine the mineralization, the analysis of calcium content was performed on 5^th^ day of culture. Trichloroacetic acid was used to remove culture medium under ultrasonication to individual hydrogel. To extract the calcium, the samples of hydrogel were incubated for 12 h at a temperature of 5 °C. Later, the extract was separated and subjected to centrifugation at 10,000 rpm for about 10 min at 4 °C temperature.

### Statistical significance

ANOVA was used under SPSS statistical software (SPSS Inc., SPSS12 for windows) to observe the statistical differences. The evaluation of statistical differences was performed by taking the significance at *p* < .05.

## Result and discussions

**Figure 1.**
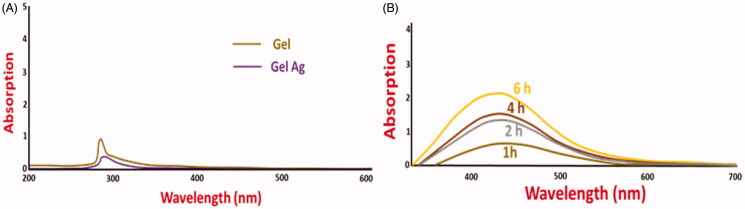
UV-vis spectra of gelatin and gelatin-AgNPs before (A) and after (B) irradiation of diffused sunlight.

Initially, the formation of AgNPs in AgNPs/Gel was confirmed visually by observing the color and characteristic features under UV-Vis spectra along with gel morphology as shown in Figure 1. Later, we examined the variation in color for both the samples after irradiated by the diffused sunlight. When silver nitrate (AgNO_3_) was added to the solution of AgNPs/Gel, it exhibited a similar pale white color when compared with the original gelatin colorless solution. However, after exposing to the diffused sunlight, the color variation was observed depending on the duration of irradiation, indicating the conversion to Ag^o^ from Ag^+^. On irradiating from 1 to 12 h, the color of the AgNPs/Gel solution changed from light yellow into darker yellow.

**Figure 2.**
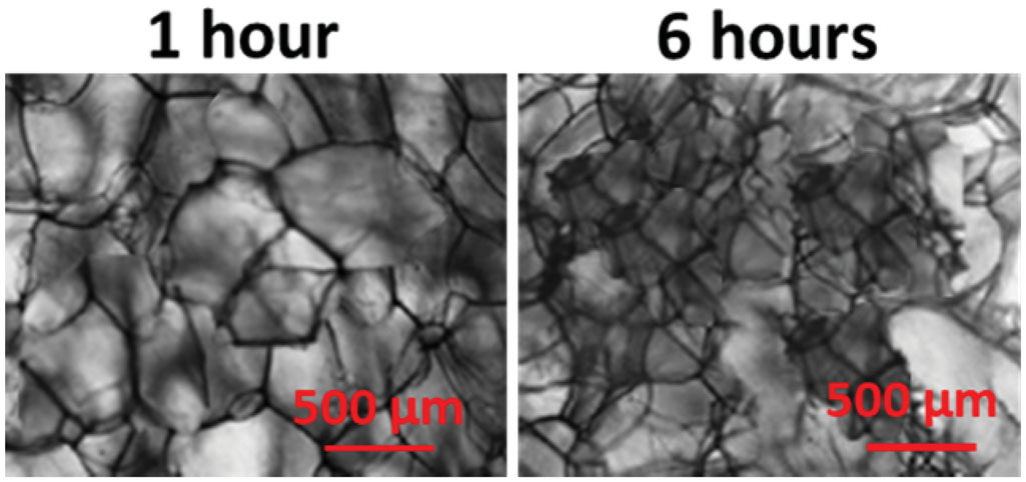
Microscopic morphology of AgNPs-Gel hydrogel.

**Figure 3.**
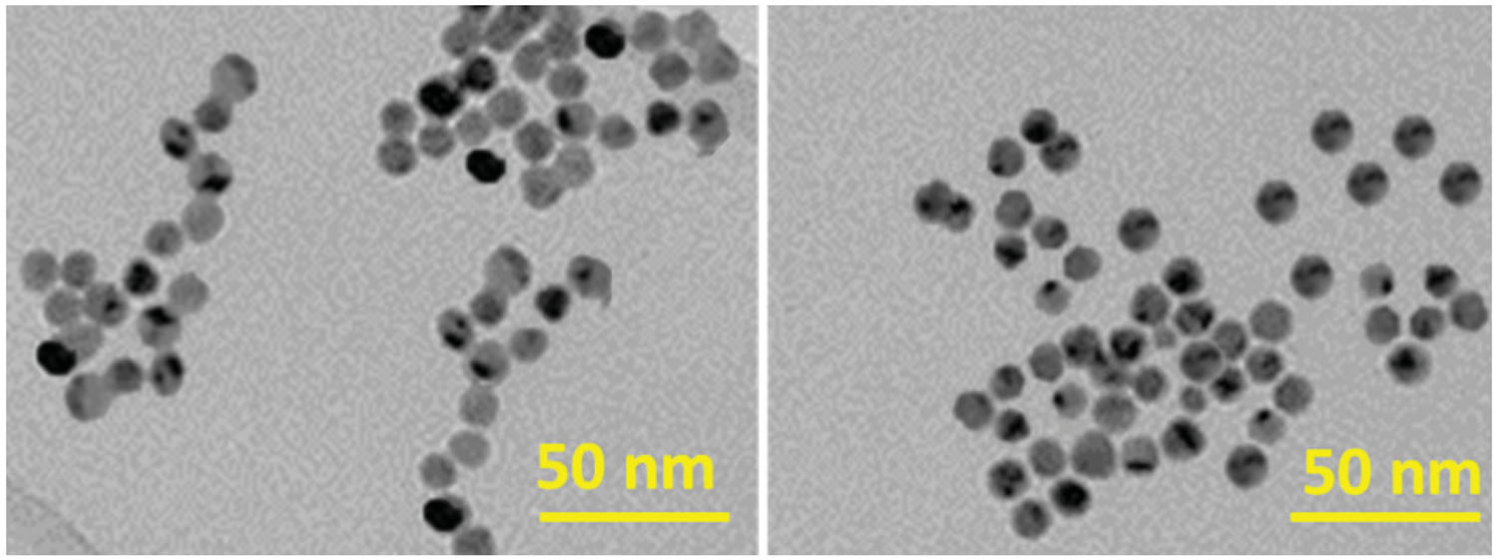
HR-TEM images of AgNPs capped with gelatin.

We also studied the morphology of the AgNPs/Gel hydrogels. From Figure 2, the AgNPs/Gel hydrogels have porous structures and no difference in the shape and size of AgNPs/Gel pores was observed. To investigate the activity of capping agent on the formation of AgNPs, the TEM analysis was performed to observe their correspondence with the above-mentioned findings, which states that various capping agents can influence the synthesized AgNPs. All together, the AgNPs/Gel hydrogels exhibited the characteristic features of mono-disperse, spherical and uniform particles (Figure 3).

### Biological studies

**Figure 4.**
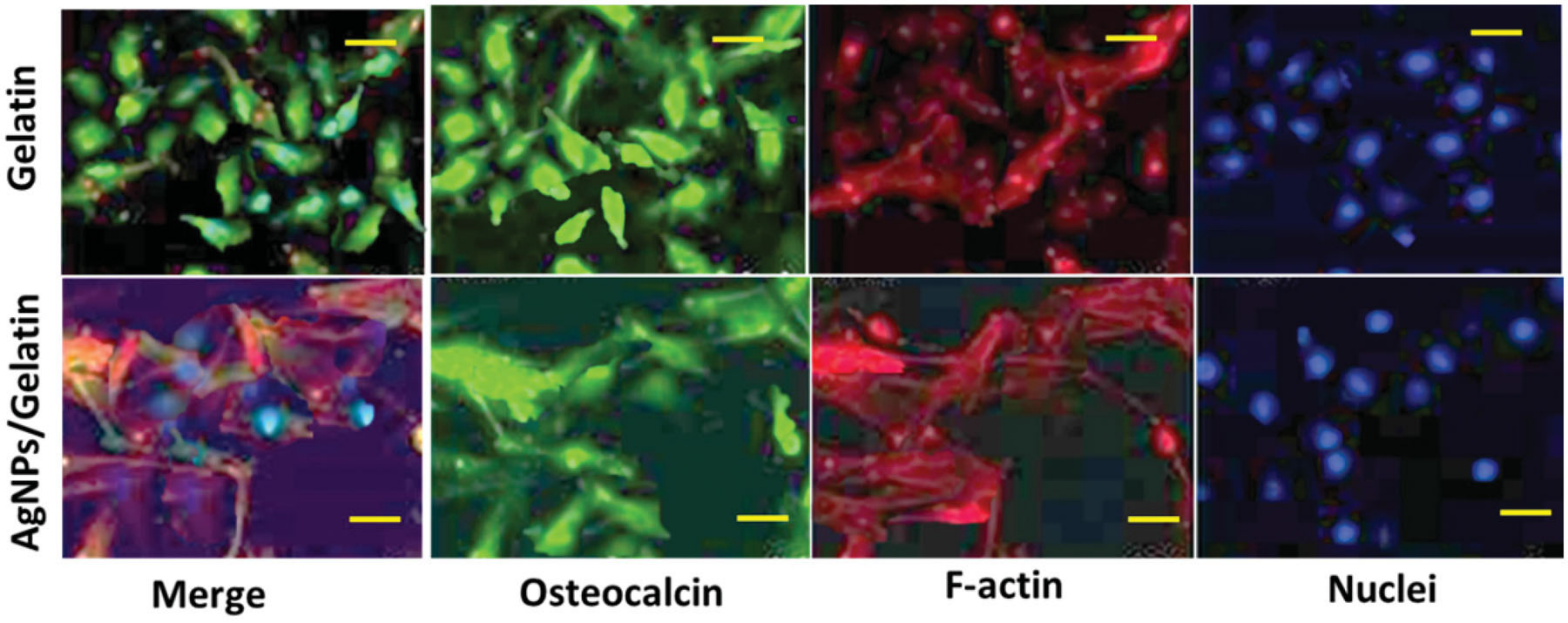
Immunocytochemical (IC) estimation for the prepared samples (AgNPs/Gel bio-composite hydrogel in the absence & presence of NPs).

The main objective of this experiment is to examine the cytotoxicity as shown in Figure 4. F- actin and osteocalcin are the early indicator for the osteogenic separation and a late marker for the osteogenic differentiation were evaluated after a period of 5 days, respectively (Bhattarai et al., 2010; Karpuraranjith & Thambidurai, 2016). The separation of stem cells over the mature osteoblasts was indicated by the presence of these markers. Gel hydrogel loaded with AgNPs exhibited higher expression of F-actin and osteocalcin proteins when compared with Gel. Hence, these findings clearly showed hydrogels with various osteogenic properties and their benefits in the application of nursing care for bone tissue engineering.

**Figure 5.**
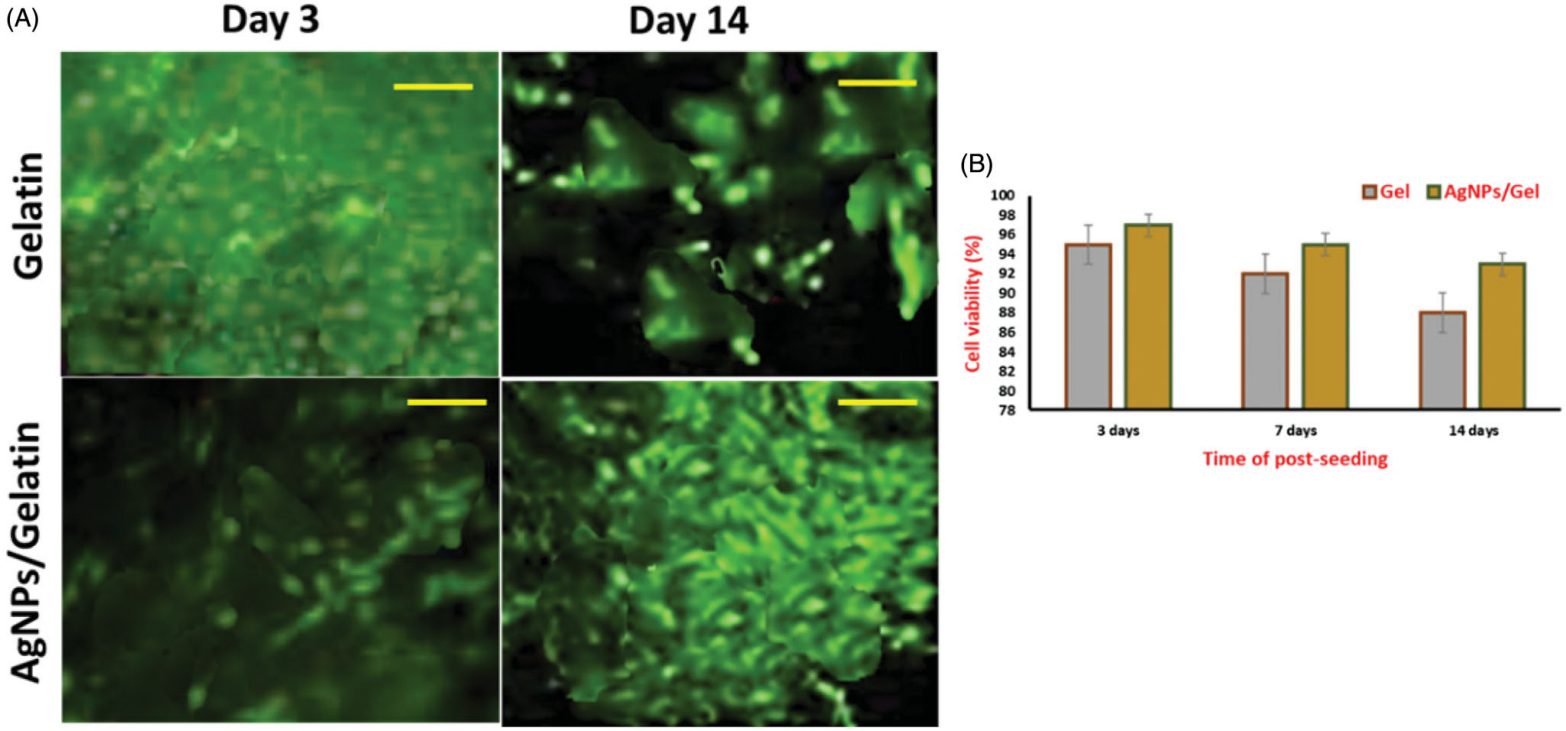
Live/dead cell fluorescent microscopic images showing cell viability after treatment with AgNPs/Gel.

The images of fluorescence spectrum of all the cell groups consisting cultures after first day and fifth day were shown in Figure 5. The red color indicates the dead cells and green color indicates the living cells. It also shows the incubation time and the live/dead (cell viability) assay of Gel and AgNPs/Gel with MC3T3-E1 cells. After the incubation period of 5 days at 37 °C, the AgNPs loaded Gel hydrogels showed low cell viability when compared to the Gel, and this could be because of the rapid degradation of hydrogel and its segments that may constitute some substance that involves in the interaction of the cell viability (Teng et al., 2010; Lu et al., 2017). Cells perform a significant role in the physiological functions on surface material; therefore, hydrogen materials exhibiting surface property will have a massive impact on biocompatibility and nontoxicity for AgNPs and Gel. From these results, it is known that the prepared AgNPs/Gel exhibited higher cell viability indicating their use in bone fracture treatment applications. However, the lower cytotoxicity of various biologically synthesized bimolecular capped AgNPs is already reported in literature (Sulaiman et al., 2015, 2014, 2012; Taha et al., 2019).

**Figure 6.**
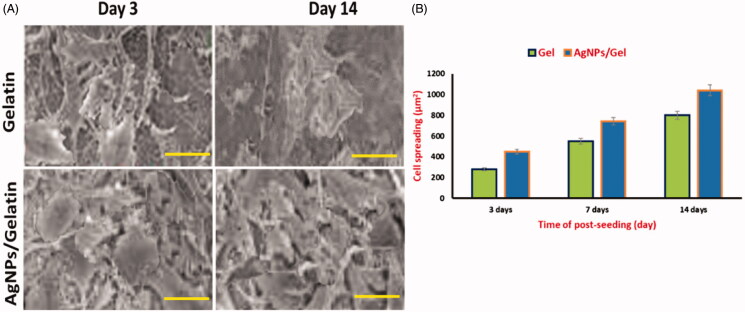
SEM microscopic images showing the cell viability upon treatment with AgNPs/gelatin.

In Figure 6, the cytocompatibility of bandages has confirmed the DAPI/Actin staining of the MC3T3-E1 cells attached onto bandages (Wu et al., 2009). On examining the fluorescence images of DAPI and Actin staining analysis after first day and fifth day, we identified that the AgNPs/Gel hydrogel bandages were having maximum cell attachment in comparison to the Gel. Therefore, the AgNPs loaded Gel hydrogels displayed a positive effect in terms of cell attachment. The percentage of cell viability apparently recovered after the seeding period of five days in AgNPs induced Gel hydrogel and gelatin, and it was observed to be 82% and 88%, respectively. After the 5th day of seeding, the cell spreading test performed and we recorded the values of Gel (480 µm^2^) and AgNPs/Gel (250µm^2^). Hence, the outcomes suggested that the AgNPs/Gel can be used for various potential applications like fracture healing and nursing care. Thus, the attained results of AgNPs loaded Gel hydrogel is better in comparison to other alternate studies like nanocomposite scaffolds of Silk fibroin (SF)/AgNPs which had an insignificant effect on the viability and cell attachment (Jegatheeswaran et al., 2016).

**Figure 7.**
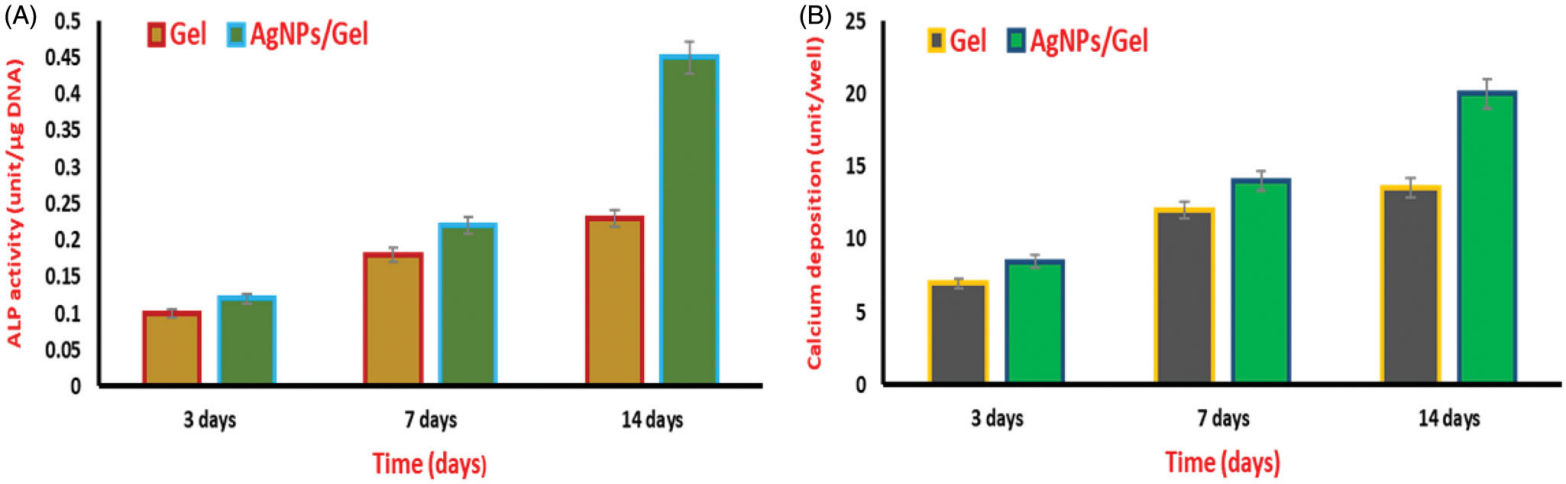
Osteoblast differentiation in-vitro upon exposure with AgNPs/gelatin. ALP activity (A) and Calcium deposition (B).

After 5 days of culture with AgNPs/Gel and gelatin hydrogel, the ALP content was examined in MC3T3-E1 cells (Figure 7). Despite the cell culture of 5 days in the hydrogel, there is no considerable difference in the ALP concentration. When compared with the Gel, the AgNPs/Gel appeared have the tendency to increase with time period of culture. Therefore, the ALP content of AgNPs/Gel hydrogel was observed to be ∼220 mg (Johari et al., 2018). The amount of calcium in MC3T3-E1 cells after culturing for five days in AgNPs/Gel Gel was shown in Figure-7B. Owing the higher mineralization, the calcium deposition content in AgNPs/Gel hydrogel was recorded to be 4.3 mg. Gel hydrogels alone were observed with low calcium content deposition (Jin et al., 2017).

## Conclusions

In conclusion, AgNPs loaded Gel hydrogels were synthesized and stabilized using gelatin. The characteristics of fabricated hydrogels were evaluated after confirming with various characterization techniques such as UV-Vis, HR-TEM analysis. Furthermore, the outcomes of cytotoxic effects in the cells verified that the AgNPs/Gel hydrogels are not harmful toward osteoblasts. The results of cell fixation after incubating for five days have demonstrated the enhanced survival of cells *in vitro* and their spreading onto the AgNPs loaded Gel hydrogels. Moreover, the bone fracture can be treated by AgNPs/Gel nanostructures, which shows its potential application in nursing care.

## Research involving Human Participants and/or Animals

No animals or human participants were involved in this study.
